# Photodynamic Therapy Healing a Refractory Radiation-Induced Ulcer on the Chest Wall Postmastectomy Radiotherapy for Breast Cancer: A Case Report and Literature Overview

**DOI:** 10.7759/cureus.67962

**Published:** 2024-08-27

**Authors:** Edward Yu, Patricia Tai, Francisco Perera, Kevin Jordan

**Affiliations:** 1 Department of Oncology, Western University, London, CAN; 2 Department of Oncology, University of Saskatchewan, Saskatoon, CAN

**Keywords:** radiotherapy, post-mastectomy, wound, non-healing, hyperbaric oxygen therapy, photodynamic therapy, cancer, breast, oncology, ulcer

## Abstract

Photodynamic therapy (PDT) has been used to treat cancers. It has also been used to treat infectious diseases and inflammatory conditions. PDT promotes wound healing, while clinical use of PDT for wound healing is uncommon and not thoroughly investigated. We report a 75-year-old female with a radiation-induced non-healing ulcer for five years on the chest wall postmastectomy radiotherapy. Biopsy showed epidermal erosion with dermal inflammation but no recurrent cancer. She was referred from the wound care clinic after multiple unsuccessful attempts to manage wound healing for two years involving daily home nursing visits. PDT was discussed with the patient who consented to PDT instead of hyperbaric oxygen therapy (HBOT) for fear of its side effects. Her wound improved after a total of three treatments and the process of wound healing continued for 14 months since her first treatment session. The presented case supports the beneficial effects of PDT on chronic ulceration impeding healing of a postmastectomy radiotherapy wound. To our knowledge, this report is unique in documenting details of PDT healing a chronic refractory ulcer of five years, which developed after cancer therapy (mastectomy and radiotherapy). Further clinical study of PDT is needed on wound healing post-surgery and radiation in cancer patients. An overview of HBOT in comparison with PDT for wound healing is presented.

## Introduction

Photodynamic therapy (PDT) is a treatment used for more than 40 years in skin conditions as follows [1]. It involves the use of light in conjunction with a photosensitizer to generate reactive oxygen species (ROS) that induce various biological reactions in treated tissues. It is an approved indication for the treatment of actinic keratosis, superficial basal cell carcinoma, and squamous cell carcinoma in situ [1]. In addition, it can lower or eradicate the bacterial load in mixed infections to stimulate skin healing and epithelialization process [2].

Managing ulcers in irradiated skin remains a challenge for oncologists. In vitro and in vivo research settings have both demonstrated the efficacy of PDT in wound healing across different types of wounds [3-6]. These studies suggested that its primary mechanism of action in wound healing involves several key aspects. Firstly, PDT stimulates the immune response by enhancing the efficacy of leukocytes in combating diseases and promoting the release of immune modulators such as interleukins and growth factors [3-6]. This modulation of the immune system contributes to tissue repair and regeneration. Additionally, PDT induces direct tumor cell death, elicits inflammatory responses, and impacts the functionality of tumor blood vessels, further promoting wound healing processes [7]. Herein, we report a clinical case of PDT used successfully in radiation-induced chronic ulcerated wound postmastectomy and radiotherapy. We discuss management alternatives for this situation and the pathophysiology augmented by PDT that leads to healing of chronic ulcers [8].

## Case presentation

In 2021, a 75-year-old lady presented to EY, the first author with a history of breast cancer treated with mastectomy and radiotherapy (megavoltage photon 50 Gy with electron boost of 10 Gy to scar to a total dose of 60 Gy in 30 treatments) to the chest wall. The isodose lines were 95% for the first 25 fractions and 85%-95% for the boost area. The boost was given due to a close resection margin. Eleven months after radiotherapy, she developed a non-healing chest wall ulcer in the boost field. She already had the ulcerated wound for five years. In the last two of these five years, she was followed in the wound care clinic and required daily home nursing services to change dressings. The lesion was biopsied. The pathology report showed epidermal erosion with superficial acute chronic dermal inflammation and focal atypia. There was no evidence of malignancy. Both the pain of the non-healing ulcer and the daily home visit by nurses for the weeping wound were affecting her quality of life. With no major improvement in the chest ulcer through the wound care clinic and limited options available, she was referred to our team for consideration of PDT by her radiation oncologist. She was otherwise healthy, only with a history of borderline diabetes being controlled with diet.

**Figure 1:**
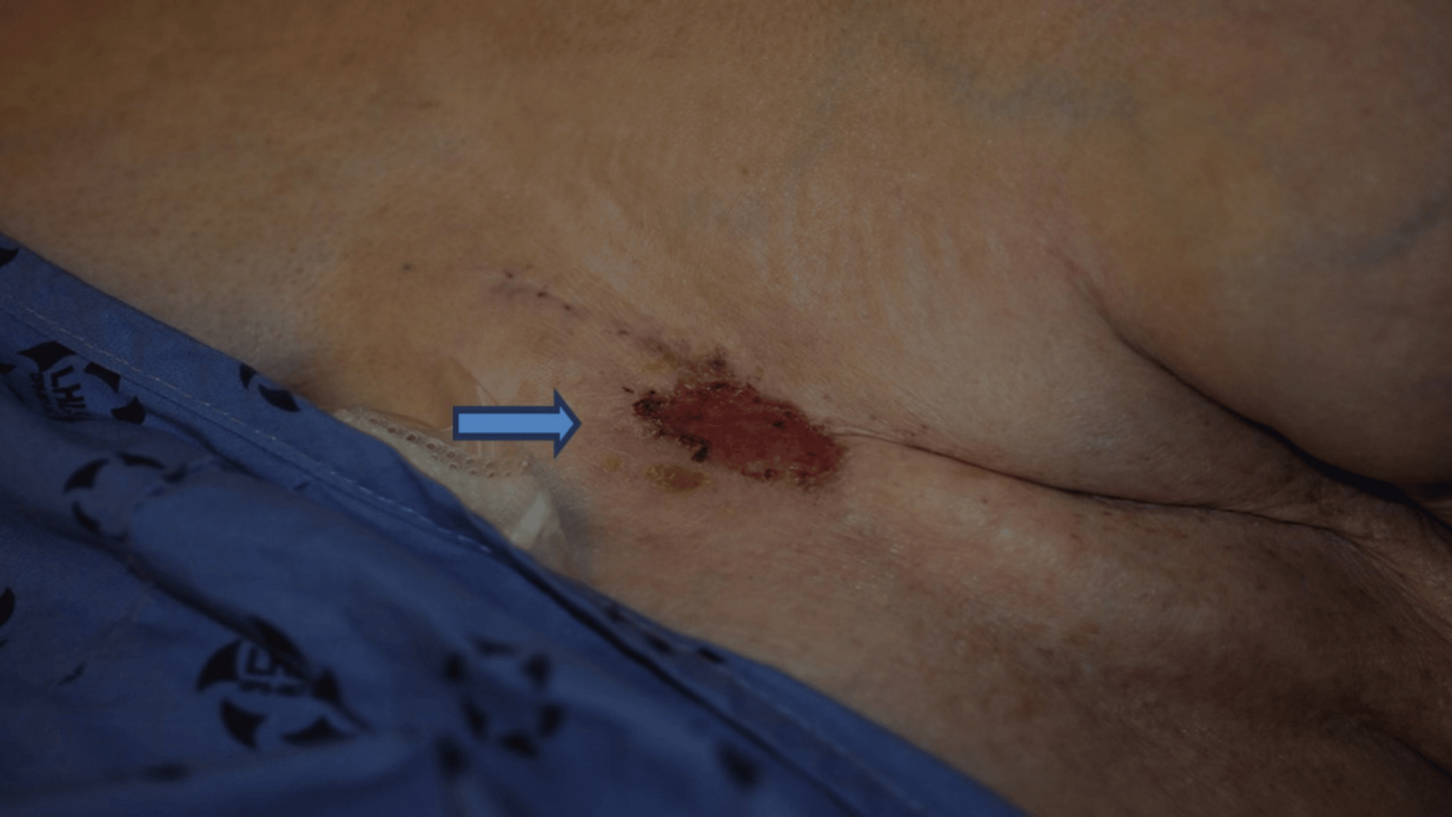
Photograph of a non-healing wound (blue arrow) for five years postmastectomy and radiotherapy to the chest wall, prior to photodynamic therapy.

On physical examination, the non-healing crusted lesion on the chest wall was within the irradiated field, immediately adjacent to the mastectomy scar. It was superficial, measuring 3.5 x 1.5 cm with central fibrotic tissue (Figure 1). There was no exposure of the underlining ribcage. There were chronic irradiation changes seen along the mastectomy scar with some telangiectasia. Mild lymphedema was seen in the ipsilateral arm.

PDT was discussed thoroughly with the patient, including alternatives. Hyperbaric oxygen therapy (HBOT) has side effects such as pain in the ear or visual changes. In addition, diabetic patients may develop hypoglycemia during HBOT. Uncommon side effects are lung trauma, seizures, lung collapse, and oxygen toxicity in susceptible individuals. She consented to receive PDT instead of HBOT for fear of its side effects. 5-Aminolevulinic acid (ALA) was mixed with Glaxal-base cream as a carrier (5% by mass). It was applied to the crusted ulcerated area, see schema in Table 1.

**Table 1:** Schema of ALA photodynamic therapy (PDT). ALA, 5-aminolevulinic acid; J/cm^2^, Joules per square centimeter; LED, light-emitting diode; mW/cm^2^, milli-Watt per square centimeter; nm, nanometer.

Steps	Materials and details
Mix ALA	With Glaxal-base cream (5% by mass)
Photo wound then apply ALA to ulcer	
Incubate	For 5 hours
Checking for red fluorescent uptake, photo	Use blue light (from a filtered actinic lamp with a peak at 420 nm)
Photodynamic therapy begins	Red (630 nm, 37 J/cm^2^) LED array lights
Fluence	A nominal fluence rate of 0.03 mW/cm^2 ^at 30 cm distance produced a fluence of 54 J/cm^2^
Duration	30-minute exposure
Total number of treatment(s) or fraction(s)	One fraction first then follow-up. Photo. May repeat PDT later

**Figure 2:**
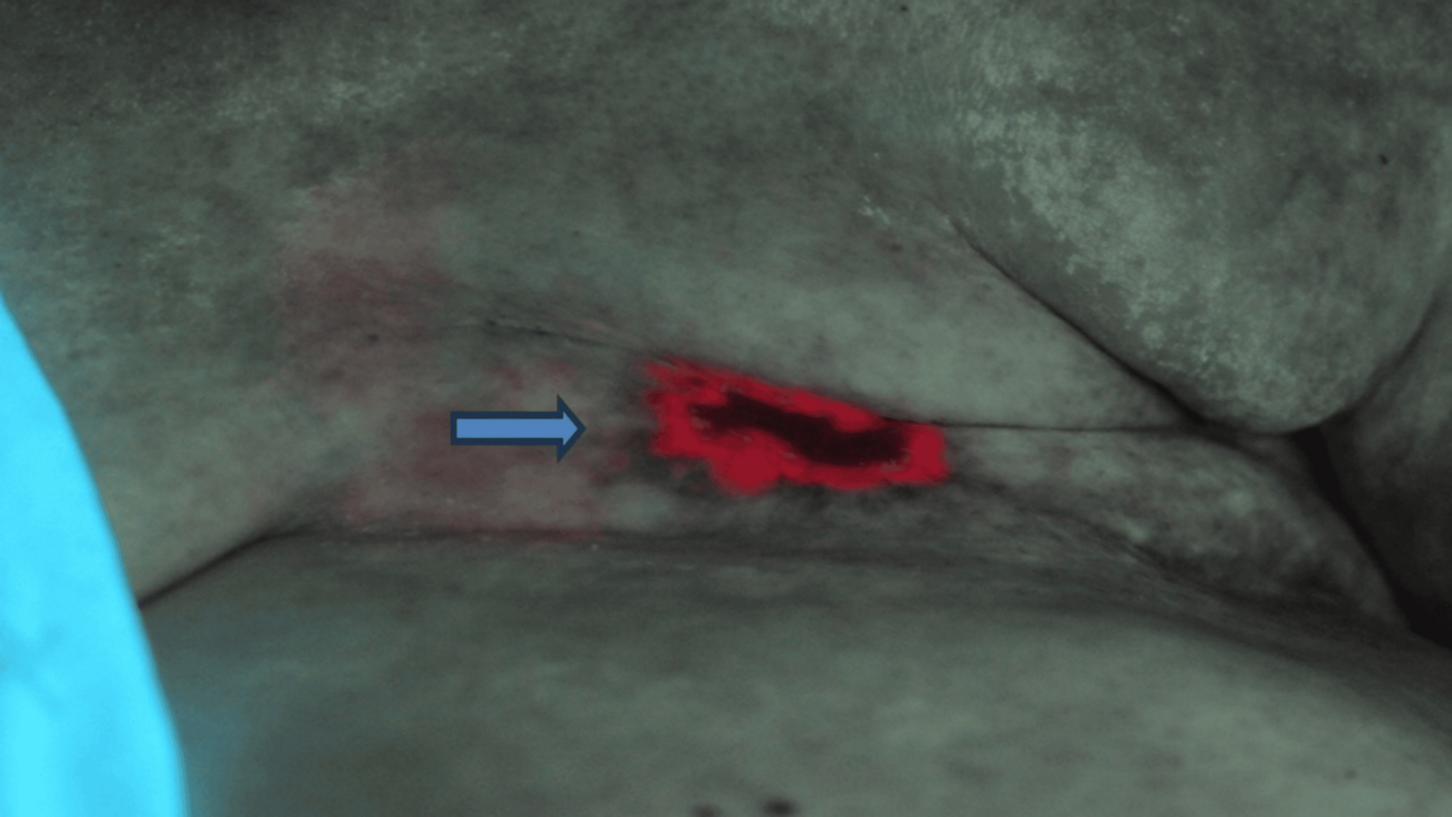
Photograph of red fluorescence at non-healing skin tissue region (blue arrow) around the central fibrotic tissue after 5-aminolevulinic acid (ALA) application and uptake with blue light excitation.

Photographs were taken to document the baseline and future treatment response. Five hours after the initial application of ALA, red fluorescence was observed around the rim of the fibrotic lesion (Figure 2) with blue light excitation.

**Figure 3:**
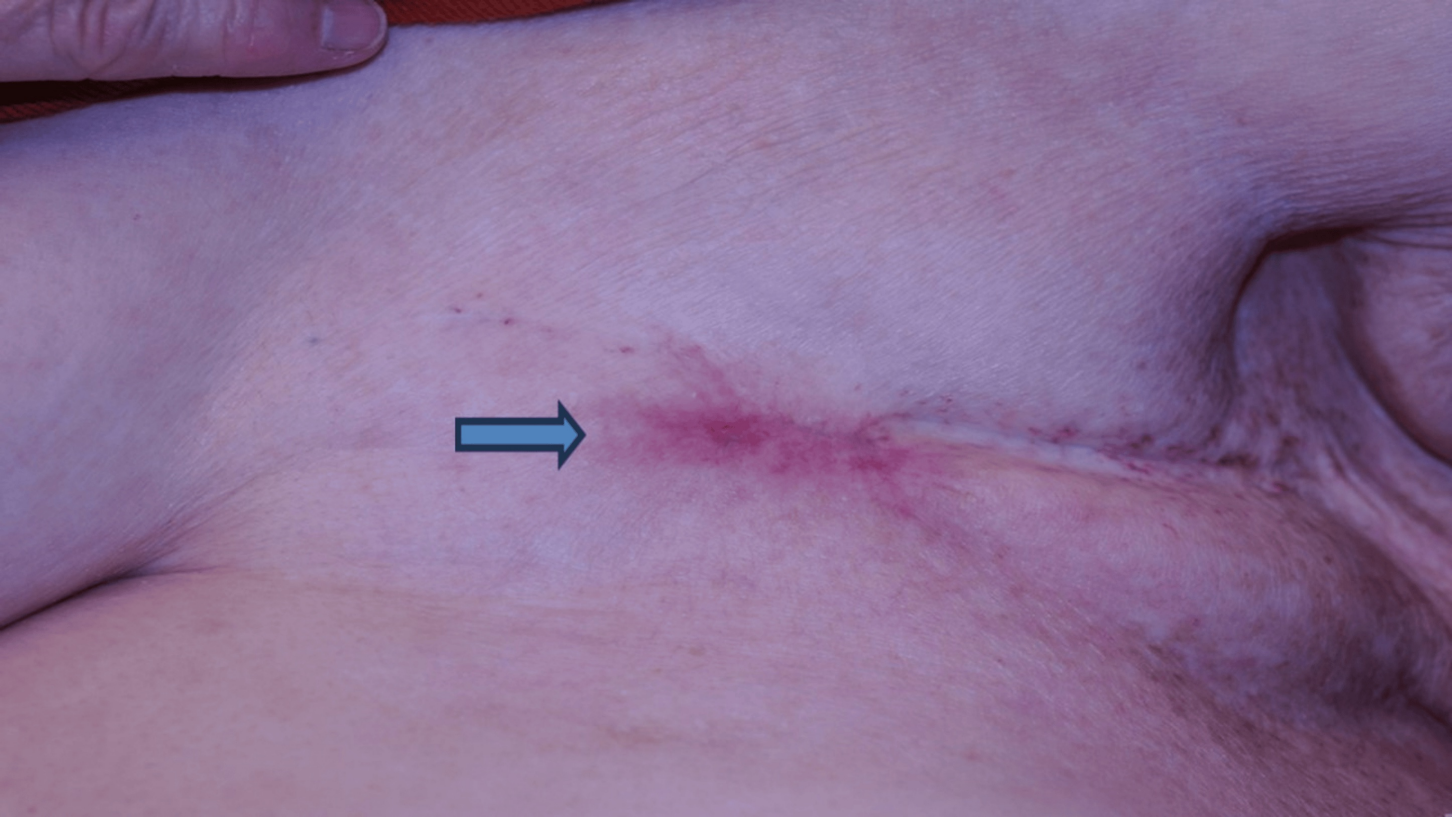
Photograph of the almost healed wound (blue arrow) three months after the first photodynamic therapy session.

The blue light was a custom filtered actinic lamp with an emission peak at 420 nm. The patient received red light treatment (630 nm, 54 Jcm^2^) for 30 minutes in one fraction with a custom light-emitting diode (LED) array light. She only had mild dermatitis with the typical ALA PDT pain during and after the session that she was able to tolerate. There was no treatment-related complication. A second PDT treatment was given four weeks later. Follow-up at three months after the first PDT showed an improvement of her wound (Figure 3).

**Figure 4:**
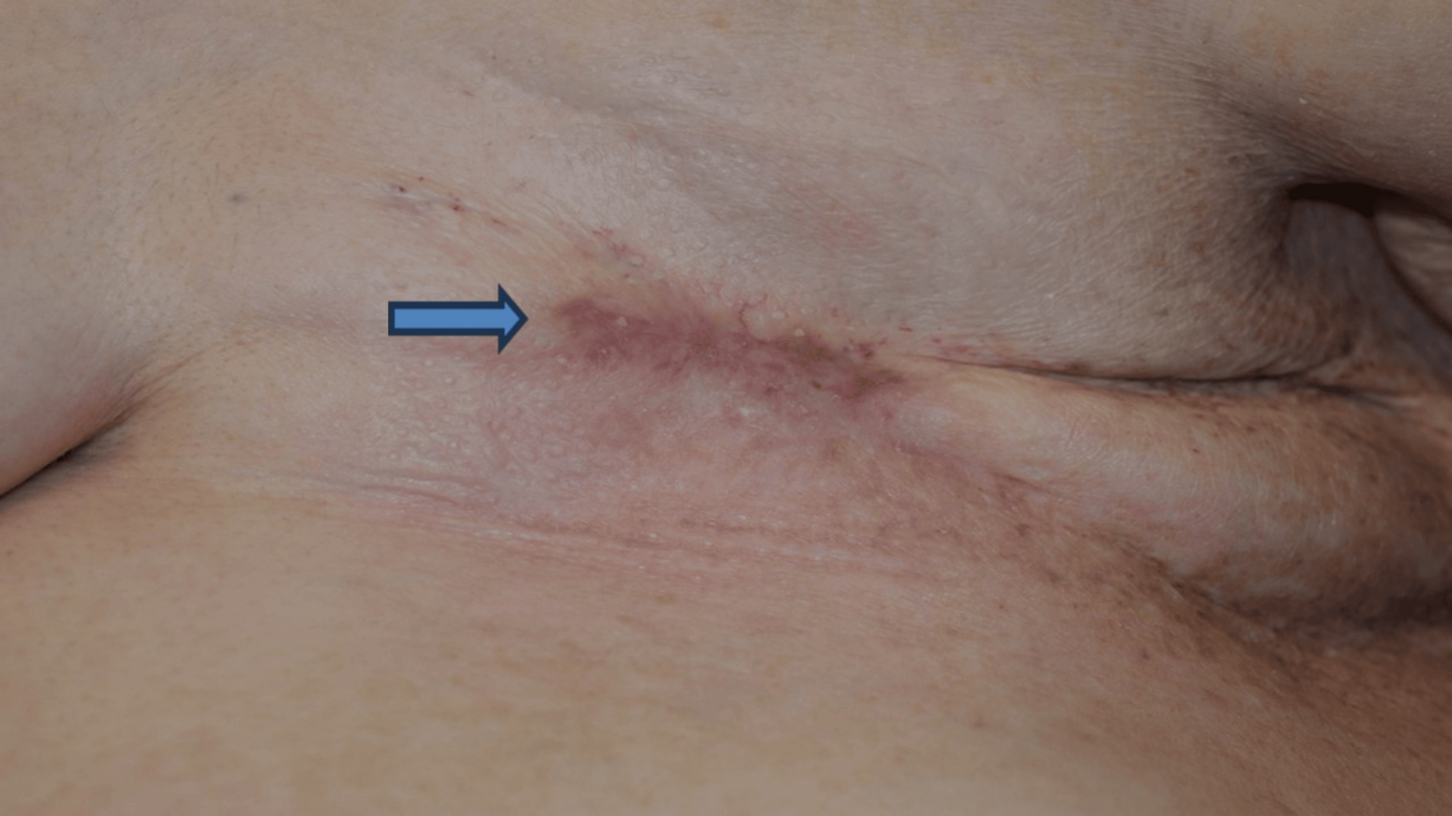
Fourteen months since the first photodynamic therapy, wound healing continued (blue arrow).

**Figure 5:**
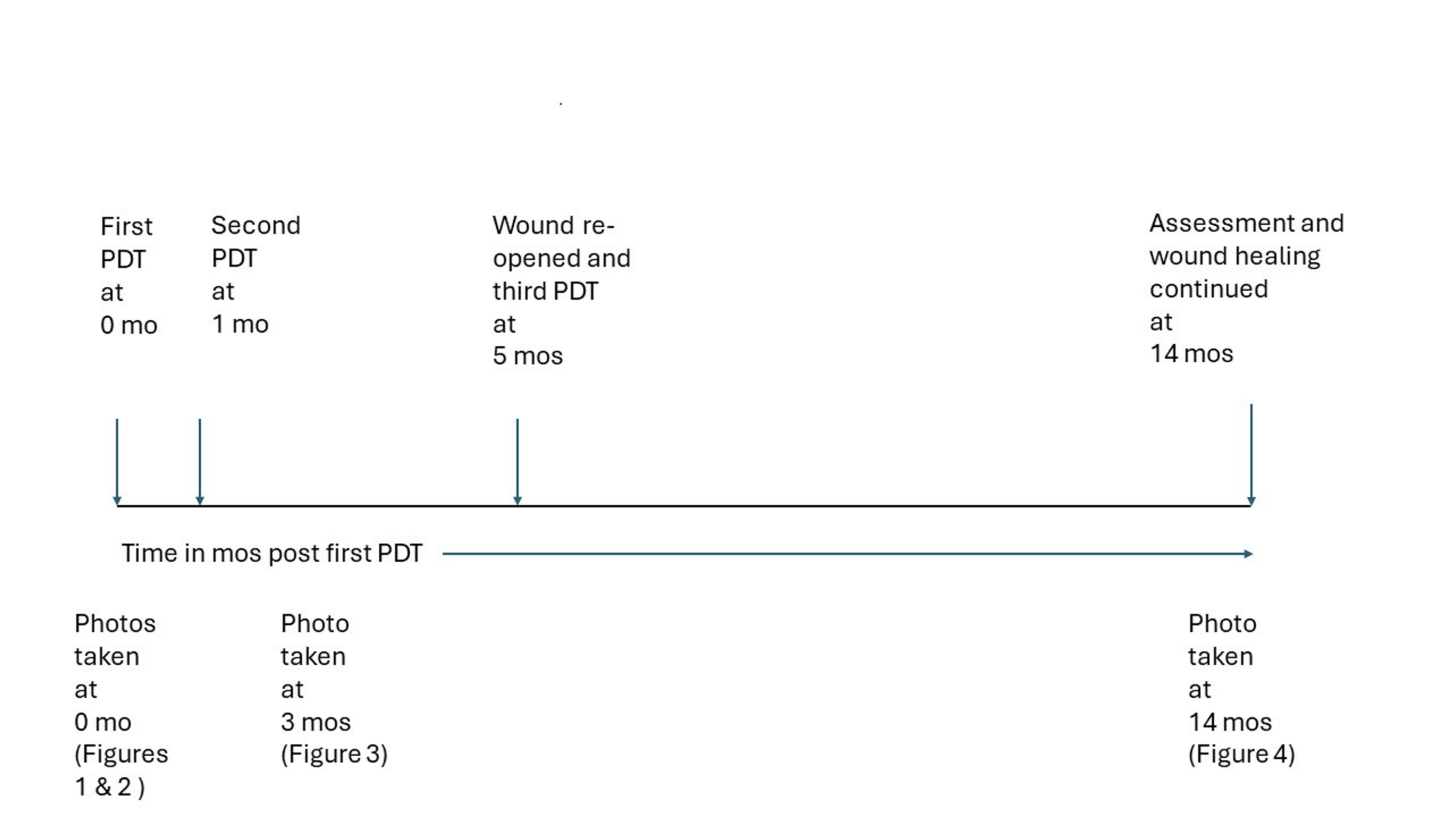
Timeline of photodynamic therapy (PDT) and follow-up in months (mos).

She returned two months later, complaining of reopening of her wound due to “subcutaneous fluid”. She was investigated with chest x-ray and chest wall ultrasound to confirm the absence of chest or subcutaneous fluid, and another treatment of PDT was given. She was reassessed at three and nine months after the third treatment with no evidence of any recurrent cancer or ulcer. The previous crusted ulcer in the chest wall continued to improve at 14 months since the first PDT treatment (Figure 4), and the timeline is summarized in Figure 5.

## Discussion

Our case report is unique to describe the PDT in detail for treating a five-year skin ulcer, although we are not the first investigators to demonstrate the benefit of PDT in treating radiation-induced skin ulcer. The exact underlying mechanism of ALA PDT for wound healing is not completely clear. In vitro, animal and clinical studies indicate that PDT can promote cell apoptosis, proliferation, migration, and adhesion [9,10]. Castano and co-workers [11] reported that wound healing was stimulated in mice 30 minutes after a single exposure to light of red (632 nm or 670 nm) or near-infrared (IR) (810nm) wavelength (either coherent laser or non-coherent LED light). The benefit from PDT in previously irradiated tissue is believed to be associated with the above phenomena of cellular microenvironment changes, promoting wound healing. ALA PDT has been utilized for wound healing in recent years and warrants more research to investigate the full mechanism of action for all its positive effects [12].

PDT plays a role in various levels (severities) of wound healing, including acute, chronic, and aging wounds. In acute wounds, studies have shown that PDT triggers apoptosis and influences the production of fibroblasts and keratinocytes, leading to the secretion of matrix metalloproteinases, cytokines, and growth factors for tissue remodeling [7,13]. In chronic wounds, investigations have demonstrated that PDT stimulates an increase in inflammatory cells such as mast cells, regulatory T cells (Tregs), and macrophages. It elevates the expression of transforming growth factor-beta (TGF-β), promoting epithelial-mesenchymal transition, and facilitating fibroblast recruitment [14,15]. Regarding aging-related skin alterations, research has shown that PDT improves skin smoothness, reduces actinic elastosis, and addresses dyspigmentation/fine lines, resulting in increases in skin thickness and collagen synthesis [16]. The abilities of PDT (to modulate cellular processes, influence immune responses, and promote tissue remodeling across different levels of wound healing) support its potential as a therapeutic intervention in dermatological applications [17].

Among many alternatives for healing radiation-induced ulcers, pentoxifylline, vitamin E, HBOT, mesenchymal stem cells, interferon-gamma, growth factors, ginkgo biloba (fruit of a plant), and surgical debridement/grafting/flap coverage have been reported. HBOT was proposed to be able to reverse radiotherapy side effects in a limited way [8,18]. Unlike PDT, HBOT is a medical treatment that involves breathing pure oxygen in a pressurized room or chamber. It is commonly used to treat various medical conditions, including radiation-induced wounds, and has been explored for its potential benefits in accelerating skin wound healing [19]. Table 2 summarizes the difference between HBOT versus PDT for wound healing.

**Table 2:** Hyperbaric oxygen therapy (HBOT) versus photodynamic therapy (PDT) in wound healing.

	HBOT	PDT
Mechanism of action	Pure oxygen administered at increased atmospheric pressure enhances tissue oxygenation, promotes angiogenesis, and supports various cellular processes for wound healing.	It utilizes a photosensitizing agent that is activated by light of a specific wavelength, to generate reactive oxygen species (ROS) that selectively damage targeted cells, such as cancer cells or bacteria.
Indications	For compromised tissue oxygenation: diabetic ulcers, arterial insufficiency ulcers, radiation-induced non-healing wounds, decompression sickness and carbon monoxide poisoning, etc.	Infected wounds or ulcers, superficial skin cancer and lung cancer, precancerous lesions, acne and bacterial skin infections.
Treatment procedure	Put patient in a pressurized chamber for 90 minutes to 2 hours. Multiple sessions are needed.	A photosensitizer is applied followed by an incubation time period. Then the lesion is exposed to light of the appropriate wavelength. Duration and number of PDT sessions depend on the specific condition.
Side effects	Generally mild: ear barotrauma (pressure-related ear discomfort), pain in ear/sinus/tooth, claustrophobia, fatigue, temporary near-sightedness and hypo-glycemia in diabetics.	Generally mild and temporary, resolving in a few days to weeks: skin redness, swelling, pain, photosensitivity.

HBOT requires capital investment of a pressurized room or chamber and special technical expertise. Only a few centers exist in Canada. The above patient lived 200 km away from such a center. Travel expenses and being away from home are inconvenient and constitute a hassle for a senior. Although HBOT was discussed with her, she was not interested due to potential side effects.

In vitro cellular and animal research of PDT on wound healing is extensive [2-5]. Clinical cases using PDT to promote or enhance wound healing for chronic radiation-induced ulcers are limited in the literature [20]. Marquitti et al. [6] reported a breast cancer patient with wound healing issue at areola-papillary complex one-month postmastectomy without irradiation. PDT was employed, and the two-month necrotic wound healed within 19 days after the start of PDT. Our patient had the radiation-induced skin ulcer for five years and failed conventional wound care support for two years. Our detailed documentation will assist clinicians when encountering similar situations. We were pleased with the result as her quality of life was impacted by the repeated infections with pain, repeated clinic follow-up, and daily home nursing visits which were burdens to the patient and the healthcare system. The lymphedema from mastectomy and radiotherapy added to her suffering. Our finding supports PDT in healing of previously irradiated wound when HBOT is not the patient’s choice.

While both HBOT and PDT can play a role in skin wound healing, they operate through different mechanisms and are used for different indications. The choice between the two treatment modalities will depend on the specific characteristics of the wound, the underlying condition, side effects of the therapy, and the patient's overall health.

We recognized the limitations of the present report being a single patient case, with relatively short follow-up time and incomplete or absent coordinated laboratory analysis on wound microbial cultures, tissue changes analysis regarding cellular angiogenesis, re-epithelization, activated inflammatory cells, macrophage and fibroblast, etc., to support healing process. This report corroborates the hypothesis that PDT improves wound healing in the literature and serves as a reminder to clinicians who run out of options for refractory radiation-induced ulcers and wounds. Further clinical trial(s) may be warranted to assist in patient’s choice of management in wound healing postcancer therapy.

## Conclusions

Our case report is unique to describe the PDT in detail for treating a five-year skin ulcer, although we are not the first investigators to demonstrate the benefit of PDT in treating radiation-induced skin ulcer. Topical ALA-induced PDT promotes healing for ulcerated wounds as a result of previous radiotherapy. It can be a potential alternative option for patients with chronic or refractory radiation-induced wounds. The concise literature overview shows that PDT is effective even for infected skin wounds with few side effects, by decreasing microbial load and repairing injured tissues.
